# Sishen Pill Treatment of DSS-Induced Colitis *via* Regulating Interaction With Inflammatory Dendritic Cells and Gut Microbiota

**DOI:** 10.3389/fphys.2020.00801

**Published:** 2020-07-09

**Authors:** Fang Chen, Yu-Ting Yin, Hai-Mei Zhao, Hai-Yan Wang, You-Bao Zhong, Jian Long, Duan-Yong Liu

**Affiliations:** ^1^Department of Postgraduate, Jiangxi University of Traditional Chinese Medicine, Nanchang, China; ^2^School of Pharmacy, Jiangxi University of Traditional Chinese Medicine, Nanchang, China; ^3^College of Traditional Chinese Medicine, Jiangxi University of Traditional Chinese Medicine, Nanchang, China; ^4^Party and School Office, Jiangxi University of Traditional Chinese Medicine, Nanchang, China; ^5^Science and Technology College, Jiangxi University of Traditional Chinese Medicine, Nanchang, China; ^6^Formula-Pattern Research Center of Jiangxi, Nanchang, China

**Keywords:** Sishen pill, inflammatory bowel disease, inflammatory dendritic cells, gut microbiota, dextran sodium sulfate

## Abstract

Sishen Pill (SSP) is a typical prescription in the pharmacopeia of traditional Chinese medicine (TCM), and is usually used to treat inflammatory bowel disease (IBD). It is known that inflammatory dendritic cells (DCs) and imbalance of gut microbiota play significant roles in the pathogenesis of IBD. However, it is not clear whether SSP can treat IBD by regulating interaction of DCs and gut microbiota. In the present study, the levels of inflammatory DCs and gut microbiota were analyzed by flow cytometry and 16S rDNA analysis. SSP relieved the pathological damage to the colon of mice with colitis induced by dextran sodium sulfate (DSS). As typical indicators of inflammatory DCs, the levels of CD11c^+^CD103^+^E-cadherin^+^ cells and pro-inflammatory cytokines [interleukin (IL)-1β, -4, -9, and -17A] were decreased in mice with colitis treated by SSP for 10 days. Simultaneously, the gut microbiota composition was regulated, and beneficial bacteria were increased and pathogenic bacteria were reduced. The results indicated that SSP regulated the interaction between inflammatory DCs and gut microbiota to treat DSS-induced colitis.

## Introduction

Inflammatory bowel disease (IBD), comprising Crohn’s disease (CD) or ulcerative colitis (UC), is a chronic and nonspecific inflammatory disease of prolonged disease duration (Ng et al., 2018). Unfortunately, the incidence is expected to rise, especially in western countries. IBD is characterized by a diverse set of clinical manifestations, including recurrent diarrhea, hematochezia, and celialgia (Rubin et al., 2019). In addition, it is regarded as a refractory disease by the WHO and currently has little chance of complete cure. IBD is thought to be triggered by imbalance of extensive gut microbiota and immune responses, which is closely related to a complex of environmental factors, dietary habits, and genetic inheritance (Nanjundappa et al., 2017). There is evidence that the immune system and gut microbiota dysbiosis may be the main contributors to IBD, although the pathogenesis remains unclear (Ferguson et al., 2016). The fecal microbiota composition in IBD patients differs from that in healthy individuals (Macfarlane et al., 2009). It is suggested that the relationship between intestinal immune homeostasis and microflora is important in disruption of the epithelial barrier in the pathogenesis of IBD (De Souza and Fiocchi, 2016). These intricate and complicated interactions may contribute to initiation and maintenance of intestinal inflammation. Hence, potential therapy of IBD may regulate immune status and gut microbiota.

Dendritic cells (DCs) are one of the most crucial players in all immune responses. A key role of DCs is to present antigens to T-helper (CD4^+^) lymphocytes in initiating and shaping immune responses. DCs can be subdivided into different subsets to satisfy various requirements. It has been proposed that DCs may benefit tolerogenic responses, while inflammatory DCs may drive protective immunity (Rutella and Locatelli, 2011). In mice with colitis, DCs activate regulatory T cells to induce interleukin (IL)-10 to improve UC (Flück et al., 2016). In lung cancer therapy, immunotherapy based on DCs and cytokine-induced killer cells has obvious efficacy (Mohsenzadegan et al., 2020). Interferon-γ *via* DCs facilitates T cell immunity to influenza A virus (Hemann et al., 2019). In contrast, the CD103^+^E-cadherin^+^ DC subset promotes chronic pathological inflammatory responses (Siddiqui et al., 2010). CD103^+^E-cadherin^+^ DCs produce pro-inflammatory cytokines to exacerbate T cell-mediated colitis.

The gut microbiota are essential in inmmune-mediated intestinal inflammation, and the relationship of microbial factors and DCs is also critical for maturation and function of the host immune system. Intestinal DCs restrict the pro-inflammatory effect of gut bacteria by effector T cells. Similarly, the gut microbiota in turn influence DCs. There was previous study showed that bacterially induced colitis may be limited by low levels of Toll-like receptor (TLR)-2 and TLR-4 in lamina propria DCs (Hart et al., 2005). The complex resident enteric microbiota alleviate immune responses by activating IL-10 production (Mishima et al., 2019). In experimental mice, intestinal DCs migrate into the liver to activate natural killer T cells to induce hepatitis *via* invasion of intestinal pathogenic bacterial antigens (Chen et al., 2014). Some bacteria probably modulate intestinal DCs *via* enhancing Th17 cell differentiation (Ivanov et al., 2008). According to these data, we conclude that the intestinal microbiota and DCs affect each other.

Sishen Pill (SSP) is a classical typical prescription in traditional Chinese medicine (TCM), which consists of Semen psoraleae, Fructus evodiae, Semen myristicae, and Schisandra chinensis, and has been used to treat colitis for thousands of years. The nine major bioactive components of SSP, deoxyschizandrin, γ-schizandrin, schizandrin, schizandrol B, schisantherin A, psoralen, isopsoralen, evodiamine, and rutaecarpine, have been analyzed by high performance liquid chromatography (HPLC) coupled with electrospray tandem mass spectrometry (ESI-MS/MS; Zhang et al., 2018). Our previous studies have shown that SSP attenuates the symptoms of trinitrobenzene-sulfonic-acid induced colitis by restraining nuclear factor-κB activation *via* inhibiting the NEMO/NLK signaling pathway (Wang et al., 2019), indicating that SSP had certain effect on colitis, but whether it affected the development of ulcerative colitis by another way has not been known. However, it is unknown whether SSP is active against experimental colitis by regulating the balance between inflammatory DCs and gut microbiota. Therefore, in this study, we explored the mechanism of SSP treated IBD by observing the changes in the interaction between inflammatory DCs and gut microbiota.

## Materials and Methods

### Drugs and Reagents

SSP (batch number: 16080053) was obtained from Tongrentang Natural Medicine Co. Ltd. (Beijing, China). The nine major bioactive components of SSP, including deoxyschizandrin (72.6 μg/g), γ-schizandrin (131.5 μg/g), schizandrin (258.0 μg/g), schizandrol B (71.2 μg/g), schisantherin A (25.1 μg/g), psoralen (131.08 μg/g), isopsoralen (1293.7 μg/g), evodiamine (22.2 μg/g), and rutaecarpine (24.0 μg/g), were analyzed by HPLC coupled with ESI-MS/MS (Zhang et al., 2018). Dextran sodium sulfate (DSS; molecular weight: 36,000–50,000 kD) was obtained from Sigma (St. Louis, MO, USA), and mesalazine (5-ASA; batch number: 170318) was from Sunflower Pharma (Jiamusi, China). Total DNA extraction kit, total RNA extraction kit, First-Stand cDNA reverse transcription kit, polymerase chain reaction kit, and primers were obtained from Shanghai Majorbio Bio-Pharm Technology Co. Ltd. (Shanghai, China). Mouse IL-4, IL-9, IL-17A, and IL-1β ELISA kits were obtained from Thermo Scientific Co. Ltd. (Thermo Fisher, Massachusetts, USA). PE anti-CD11c, AF647 anti-CD103, APC anti-TNF-α, and FITC anti-E-cadherin for flow cytometry were purchased from BD Biosciences (Franklin Lakes, NJ, USA).

### Animals and Treatment

A total of 32 male BALB/c mice (18–20 g, age of 4 weeks) with the animal license number SCXK (Xiang) 2016-0002, were purchased from Hunan SJA Laboratory Animal Co. Ltd. (Hunan Province, China). Throughout acclimatization and the study, experimental protocols were in accordance with the guidelines of the animal center and were approved (approval No. JZ 2018-109) by the Institutional Animal Care and Use Committee (IACUC) of Jiangxi University of Traditional Chinese Medicine, Nanchang, China. All mice were bred and maintained under specific pathogen free conditions, at 19–23°C and 35–55% humidity. The animal room was artificially alternated between 12 h light and 12 h darkness. Mice were housed in cages and had free access to distilled water and food except in exceptional circumstances. The experimental animals were fed 3 days in order to adapt to the environment and diet before the study started.

**Figure 1:**
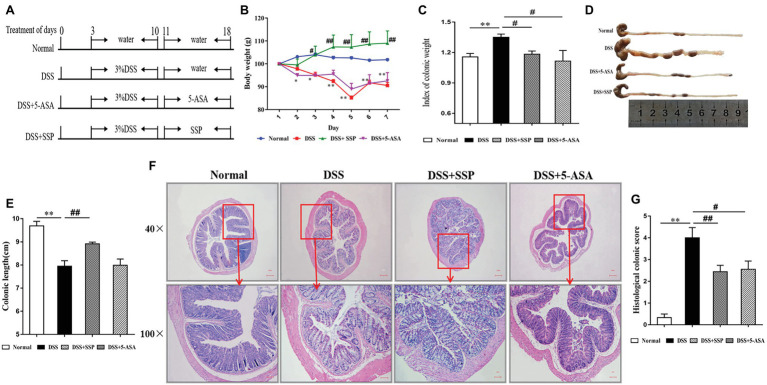
Effects of Sishen pill (SSP) administration on dextran sodium sulfate (DSS)-induced colitis in mice. **(A)** Experimental design. Relative changes in **(B)** body weight, **(C)** index of colonic weight, **(D)** macroscopic view of opened colon, **(E)** colonic length, **(F)** histological analysis (bar = 100 and 10 μm) of treated mice, and **(G)** histological colonic score. Data are presented as means ± SEM (*n* = 8). ^**^p < 0.01 versus normal group; ^#^*p* < 0.05 and ^##^*p* < 0.01 versus DSS group.

Three days later, all mice were randomly divided into four groups (eight per group): (1) normal, kept fed with water as usual and were not treated with DSS; (2) model (DSS) group, treated with DSS to induce experimental colitis without drug treatment; (3) DSS+SSP group, DSS colitis treated with SSP; and (4) DSS+5-ASA group, DSS colitis treated with 5-ASA. The latter three groups all drank 3% DSS (w/v) solution freely for 7 days. At the same time, the solution was replaced fresh every other day. On day 11 (Figure 1A), the DSS+SSP and DSS+5-ASA groups were treated with SSP (2.5 g/kg/day) or 5-ASA (0.3 g/kg/day), which was diluted in physiological saline solution for 1 week. An equivalent volume of physiological saline solution was administered to the other two groups for 7 days.

### Macroscopic Evaluation

The mice were weighed before anesthesia, and the colons were quickly removed. The colon length and weight were measured, and the colon weight index (CWI), CWI = colon weight/body weight × 100% was calculated.

### Pathological Histology Analysis

On day 18 (Figure 1A), the terminal colon tissues were excised from mice after death. The tissues were washed with phosphate-buffered saline (PBS, pH 7.2), frozen at −80°C and fixed using 4% paraformaldehyde overnight. Tissues were dehydrated in a graded ethanol series, watered for 5 min, embedded in paraffin, and cut into 4-μm-thick sections. The sections were stained with hematoxylin and eosin and observed under a light microscope for histopathological evaluation. The CWI was calculated by dividing the weight by the length. Histopathological injury scores were calculated according to inflammatory cell infiltration and tissue damage. The scoring of inflammatory cell infiltration was evaluated as 0 (rare inflammatory cells in the lamina propria) to 3 (transmural extension of the infiltration of inflammatory cells), and tissue damage was evaluated ranging from 0 (no mucosal damage) to 3 (extensive mucosal damage and extension through deeper structures of the bowel wall) (Schmidt et al., 2010).

### Elisa

Colon tissue (0.1 g) was collected, weighed, and immersed in 900 ml normal saline, followed by ultrasonic trituration and centrifugation at 3,000 rpm for 15 min in order to obtain colon tissue homogenate. ELISA was performed to detect the levels of IL-1β, IL-4, IL-9, and IL-17A. After terminating the reaction with a stop solution, the absorbance was detected using a spectrophotometer at a wavelength of 450 nm.

### Flow Cytometry

Peripheral whole blood was collected in anticoagulant tubes. Cells were isolated from the peripheral blood mononuclear cells for further analysis. The cell samples were resuspended in RPMI-1640 and incubated at 37°C in 5% CO_2_ for 3 h. The cells were fixed and permeabilized with a Cytofix/Cytoperm Kit (BD Biosciences) prior to the standard surface and intracellular staining procedures. Subsequently, cells were incubated with antibodies for 40 min at 4°C, shielded from light for surface marker staining, and then washed with PBS. Finally, cells were subjected to flow cytometry (Fortessa Flow Cytometer; Becton Dickinson San Jose, CA, USA) to analyze the final samples and exclude nonviable cells. All data were analyzed using FlowJo 7.6.1 software.

### DNA Extraction and Bioinformatic Analysis by 16S rRNA Gene Sequencing

The stool samples were collected on day 18 and immediately stored at −80°C for bacterial DNA extraction. Genomic DNA was extracted from digesta using a QIAamp DNA Stool Mini Kit (Qiagen, Valencia, CA, USA). The concentration and purification of bacterial DNA were determined by spectrophotometry. The distinct V3–V4 region of the bacterial 16S ribosomal RNA (rRNA) gene was amplified to evaluate bacterial diversity using PCR with two specific primers 515F (5'-GTGCCAGCMGCCGCGGTAA-3') and 806R (5'-GGACTACHGGGTWTCTAAT-3'). PCR products were final detected using 2% denaturing agarose gels. The resulting products were purified with an AxyPrep DNA Gel Extraction Kit (Axygen Biosciences, Union City, CA, USA) to remove unqualified products and quantified by Quanti Fluor™-ST (Promega, Madison, WI, USA). The high-throughput sequencing was combined and subjected to sequencing on an Illumina MiSeq platform (Illumina, San Diego, CA, USA) for paired-end reads. In addition, the clustering sequences were binned into operational taxonomic units (OTUs) with 97% similarity cutoff using UPARSE version 7.1. Shannon and Simpson Indexes were used to evaluate species diversity.

### Statistical Analysis

Data are presented as arithmetic mean ± SEM for each treatment group. The effect of treatments was determined by one-way ANOVA and differences between treatments were analyzed *post hoc* by Tukey’s honest significant difference test. *p* < 0.05 was considered statistically significant. All statistical tests were performed using SPSS version 17.0 (SPSS, Inc., Chicago, IL, USA).

## Results

### SSP Relieved Pathological Injury in Mice With DSS-Induced Colitis

Experimental colitis was established in mice and treated as shown in Figure 1A. The typical symptoms of experimental colitis were weight loss, diarrhea, and shortened colon length. Mice exposed to DSS developed colitis and clinical symptoms that persisted for about 10 days. Compared with the normal group, body weight (Figure 1B), and colon length (Figures 1D,E) in the DSS group were decreased. Meanwhile, the index of colon weight (Figure 1C) in the DSS group was increased compared with that in the normal group. Weight loss, colon shortening, and the increased index of colonic weight were suppressed after treatment with SSP or 5-ASA, especially in the SSP group (Figures 1B–E,G, respectively). Histopathological analysis (Figure 1F) of the distal colon of the mice treated with DSS showed severe mucosal inflammatory injury with typical changes, such as distorted crypts, edema in the intestinal epithelial layer, loss of goblet cells, inflammatory cell infiltration, and ulceration were significantly severe. The histopathological scores (Figure 1G) in the DSS+SSP and DSS+5-ASA groups were decreased when compared with those in the DSS group. As shown in Figure 1F, SSP supplementation was shown to maintain ed. the intact of the colonic mucus compared with other three groups. In addition, the mixed necrotic material in the enteric cavity and slight mucinous degeneration, but no ulcer, was noticeable in the colonic tissue collected from the SSP-treated group. 5-ASA was shown to maintain the intact of intestinal epithelial cell (Figure 1F). However, a moderate infiltration of inflammatory cells was noticed in the 5-ASA treated group. Accordingly, there is no significant difference of histology scores between SSP‐ and 5-ASA-treated groups. The above results showed that SSP was effective for treatment of experimental colitis.

### SSP Inhibited Expression of Pro-Inflammatory Cytokines

**Figure 2:**
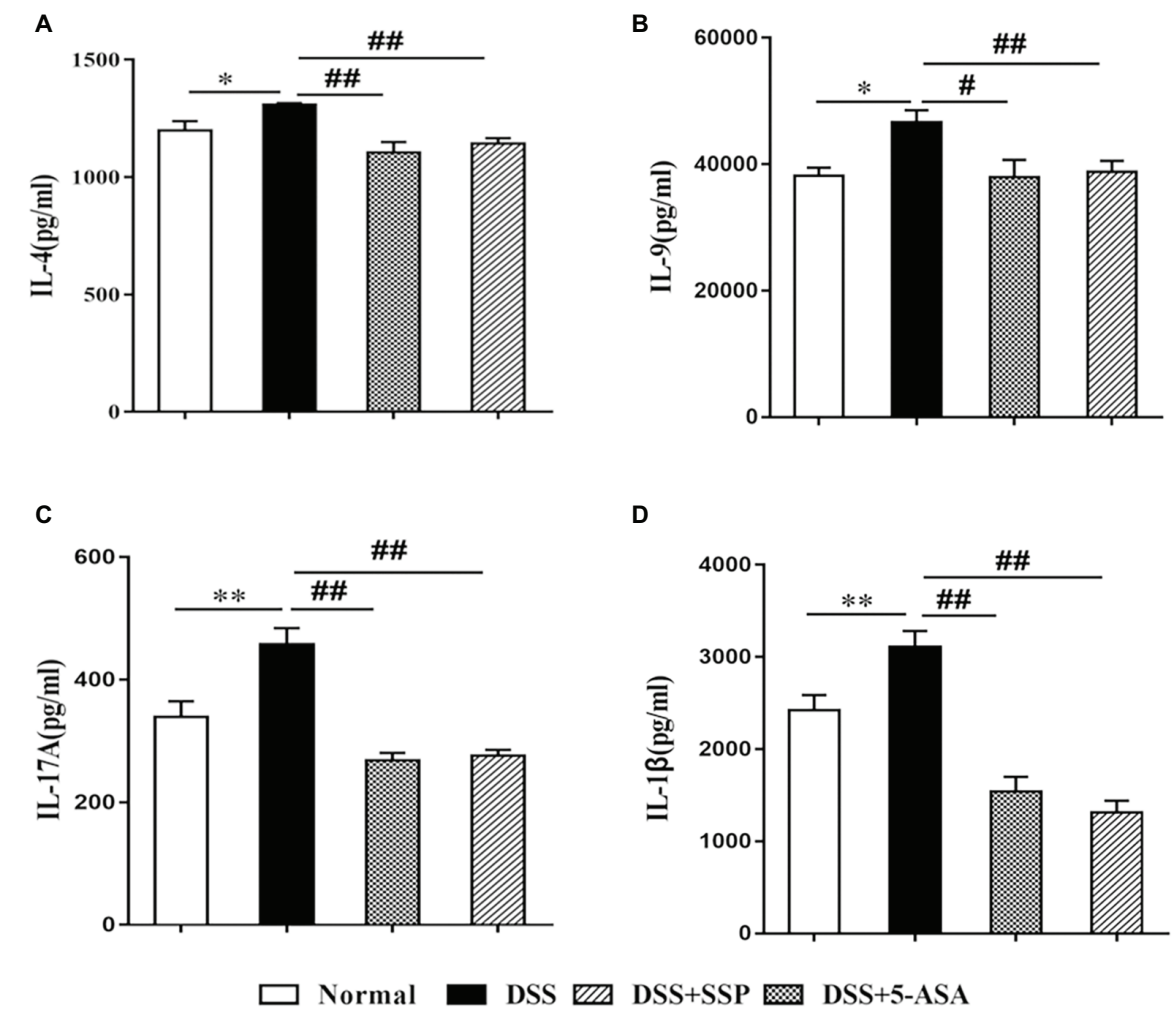
Expression of inflammatory factors in colonic mucosa in mice with colitis. **(A)** interleukin (IL)-4, **(B)** IL-9, **(C)** IL-17A, and **(D)** IL-1β. Data are presented as means ± SEM (*n* = 8). ^*^p < 0.05 and ^**^p < 0.01 versus normal group; ^#^*p* < 0.05 and ^##^*p* < 0.01 versus DSS group.

High expression of inflammatory cytokines is an important characteristic of IBD. IL-4 and IL-1β promote secretion of IL-9 that induces colonic inflammatory injury in IBD. IL-17A, a signature cytokine of Th17 cells, is a major pro-inflammatory cytokine in the pathological process of IBD (Figueredo et al., 2017). Compared with the DSS group, expression of pro-inflammatory cytokines (IL-1β, IL-4, IL-9, and IL-17A) in the other three groups was decreased (Figures 2A–D, respectively).

### SSP Restrained the Level of Inflammatory DCs

**Figure 3:**
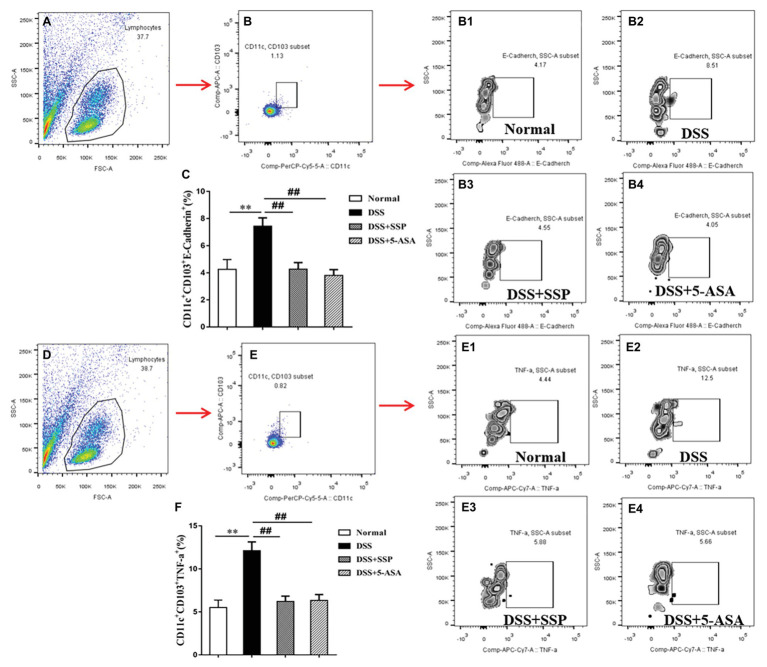
Flow cytometric analysis of inflammatory dendritic cells (DCs). **(A)** Total number of lymphocytes in the representative sample. **(B)** CD4^+^CD103^+^ cells in the representative sample. **(B1)** CD11c^+^CD103^+^E-cadherin^+^ cells in the normal group, **(B2)** CD11c^+^CD103^+^E-cadherin^+^ cells in the DSS group, **(B3)** CD11c^+^CD103^+^E-cadherin^+^ cells in the DSS+SSP group, and **(B4)** CD11c^+^CD103^+^E-cadherin^+^ cells in the DSS+5-ASA group. **(C)** Number of CD11c^+^CD103^+^E-cadherin^+^ cells in four groups. **(D)** Total number of lymphocytes in the another representative sample. **(E)** CD4^+^CD103^+^ cells in the another representative sample. **(E1)** CD11c^+^CD103^+^TNF-α^+^ cells in the normal group, **(E2)** CD11c^+^CD103^+^TNF-α^+^ cells in the DSS group, **(E3)** CD11c^+^CD103^+^TNF-α^+^ cells in the DSS+SSP group, and **(E4)** CD11c^+^CD103^+^TNF-α^+^ cells in the DSS+5-ASA group. **(F)** Number of CD11c^+^CD103^+^TNF-α^+^ cells in four groups. Data are presented as means ± SEM (*n* = 8). ^**^p < 0.01 versus normal group; ^##^*p* < 0.01 versus DSS group.

Inflammatory DCs are increased in colitis and trigger intestinal inflammation by providing antigens to T cells (Chen et al., 2019). IBD can be alleviated by inhibiting CD11c^+^CD103^+^E-cadherin^+^ cells (Niess, 2009). As the two classic inflammatory DCs promote colitis (Eberhardson et al., 2019), we revealed that expression of CD11c^+^CD103^+^E-cadherin^+^ and CD11c^+^CD103^+^TNF-α^+^ cells was increased in mice with DSS-induced colitis. The number of these cells was markedly down-regulated in colitis mice treated with SSP and 5-ASA (Figures 3A–F, respectively). These results suggest that SSP can effectively treat DSS-induced colitis by inhibiting inflammatory DCs.

### SSP Improved Composition of Gut Microbiota in DSS-Induced Colitis

**Figure 4:**
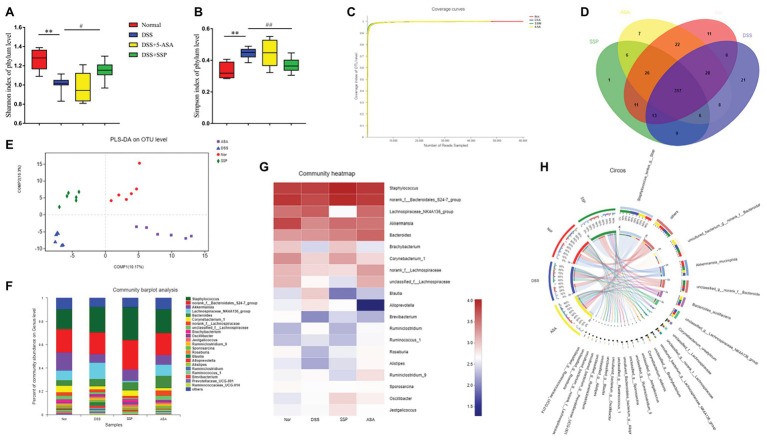
Structural comparison of fecal microbiota. Box plots indicate microbiome diversity differences of **(A)** Shannon diversity, and **(B)** Simpson diversity among four groups. **(C)** Coverage curve reveals that the coverage rate of sequencing of the four groups was high. **(D)** The Venn diagram depicts operational taxonomic units (OTUs) that differed in each group. **(E)** Partial least squares discriminant analysis (PLS-DA) score was different in each group. **(F)** SSP treatment changed the microbial community at the genus level (bar plot). **(G)** Heatmap comparing expression of different taxa between fecal microbiota. **(H)** Circos analysis showed the corresponding abundance relationship between samples and bacterial communities. Data are presented as mean ± SEM (*n* = 8). ^**^p < 0.01 versus normal group; ^#^*p* < 0.05 and ^##^*p* < 0.01 versus DSS group.

16S gene sequencing was used to compare fecal microbial populations of mice in the normal, DSS, DSS+SSP, and DSS+5-ASA groups. The Shannon Index showed that microbial diversity was reduced significantly by DSS treatment, and significantly increased in the DSS+SSP group (*p* < 0.05, Figure 4A). The Simpson Index in the DSS group was significantly higher than that in the normal and DSS+SSP groups (*p* < 0.01, Figure 4B). The coverage rate of sequencing of the four groups was high, so the sequencing results indicated that most of the diversity was captured in all samples (Figure 4C). It was suggested that the species richness in the DSS group was lower. From these analyses, the biodiversity of the DSS group was reduced after induction of colitis but restored by SSP.

Three hundred and seventeen OTUs overlapped among the groups: 364 OTUs were present in both the normal and DSS groups; 359 in the DSS and DSS+5-ASA groups; and 346 in the DSS and DSS+SSP groups (Figure 4D). Despite significant inter-individual variation, partial least squares discriminant analysis (PLS-DA) revealed that the gut microbiota in the DSS and DSS+SSP groups were separated from that of the normal group, whereas the distance between the DSS+SSP and normal groups was less than that between the DSS and normal groups (Figure 4E). Many previous studies have found structural dysbiosis in IBD. In our study using histograms, which reflected the community structure of the gut microbiota, we investigated the microbial species and their relative abundance. We conducted bar plot, heatmap, and Circos analyses to illustrate the differences in the microbiota composition among the four groups of mice. The bar plot roughly indicated that the relative abundance of different genera varied among samples at the genus level. As shown in Figure 4F, all samples contained *Akkermansia*, *Lachnospiraceae_NK4A136_group* and *Corynebacterium* at the genus level. There was a marked decrease in *Akkermansia* and *Corynebacterium* in DSS-treated mice without treatment compared to the normal, DSS+SSP, and DSS+5-ASA groups, while *Lachnospiraceae_NK4A136_group* in the DSS group was more abundant than in the other three groups (Figure 4F). We generated a heatmap showing 20 genera with a high frequency and their relative abundance. *Akkermansia* spp. and *Corynebacterium* spp. were increased after DSS colitis and mice colitis was treated with SSP (Figure 4G). This was accompanied by a decrease in the relative abundance of the *Lachnospiraceae_NK4A136_group* (Figure 4G). Circos analysis confirmed the bar plot analysis and heatmap results (Figure 4H). *Akkermansia* was increased from 18 to 24% and *Corynebacterium* from 18 to 29% by SSP treatment. However, *Lachnospiraceae_NK4A136_group* was decreased, with an abundance of 71% in the DSS group and 1.8% in the DSS+SSP group (Figure 4H). The above results showed that SSP regulated the structure of gut microbiota in DSS-induced mice colitis.

### RDA/CCA Analyze and Key Phylotypes of Gut Microbiota Altered by SSP in Colitis Mice

**Figure 5:**
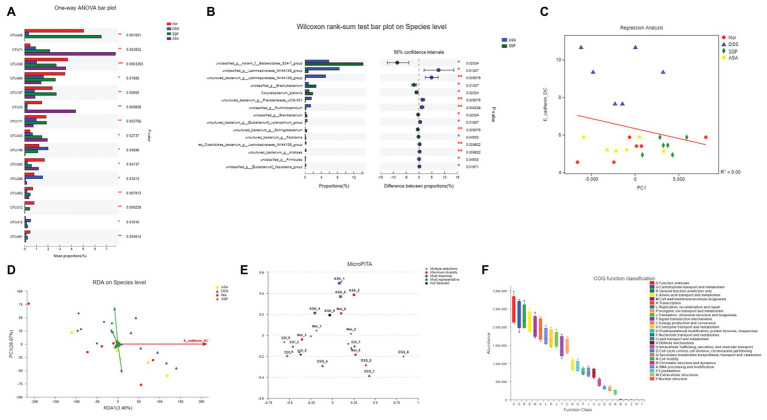
RDA/CCA analyze and species differences of gut microbiota. **(A)** Statistical comparison of the relative abundance of gut microbiota among the four groups at OTU level. **(B)** Phylotypes significantly different between SSP and DSS groups at species level. **(C)** Linear regression analysis of relationship between CD11c^+^CD103^+^E-cadherin^+^ cells and gut microbiota. **(D)** RDA/CCA ordination plots to show the correlations between bacterial community structures and inflammatory dendritic cells. **(E)** Representative microbial community comprising 24 samples; selections indicate correct example identifications of samples enriched for: maximum diversity, dissimilar, and representative. **(F)** Microbial functional features in the four groups.

To identify how to influence bacteria associated with colitis alleviation by SSP treatment, we analyzed species differences to compare gut microbiota. DSS treatment decreased several OTUs, such as OTU 23, OTU 159, and OTU 289, and increased OTU 446, OTU 71, OTU 157, OTU 171, and OTU 433 (Figure 5A). We compared the microbiota in the DSS+SSP and DSS groups at species level. In the excrement of untreated DSS colitis mice, the percentages of *Bacteroidales* and *Brachybacterium* were higher (*p* < 0.05), and *Lachnospiraceae_NK4A136_group* was lower than in colitis mice treated with SSP (*p* < 0.01, Figure 5B). Figure 3 shows that the number of inflammatory DCs was increased overall in experimental colitis. To investigate the regulation of inflammatory DCs and the improved composition of gut microbiota in experimental colitis treated by SSP, we took the CD11c^+^CD103^+^E-cadherin^+^ cells as a reference point and analyzed inflammatory DCs in relation to fecal bacteria. Inflammatory DCs were positively correlated with the DSS group, but negatively correlated with the normal, DSS+SSP, and DSS+5-ASA groups by the regression analysis (Figure 5C). The number of inflammatory DCs in the DSS group was significantly different from that in the other three groups. Thus, the cells were significantly correlated with the composition of the microbial community, suggesting that specific bacteria may affect the function of DCs. In order to find the potential correlations between the distribution of bacterial and the environmental factors, redundancy analysis (RDA)/canonical correspondence analysis (CCA) test was conducted based on the bacterial 16S rRNA gene sequences. It was found that the inflammatory dendritic cells (as CD11c^+^CD103^+^E-cadherin^+^ cells) appeared to be the most significant of the investigated factors with respect to the bacterial structures in the examined samples (Figure 5D). On the species level, the inflammatory DCs were down-regulated and shown negative correlation with SSP treatment when they were compared to the model group. The microPITA analysis showed that 24 samples from four groups were analyzed and selected by three screening methods (Figure 5E). Ten samples of DSS-6, DSS-2, SSP-5, Nor-1, SSP-3, ASA-3, Nor-2, SSP-6, Nor-4, and DSS-3 were selected by most representatives, which were the most representative core samples (Figure 5E). Another 10 samples, ASA-1, ASA-4, ASA-5, DSS-1, DSS-2, DSS-4, DSS-6, Nor-1, SSP-1, and SSP-5 were the most dissimilar (Figure 5E). The highest alpha diversity samples were obtained from ASA-2, ASA-3, DSS-5, DSS-6, Nor-2, Nor-3, Nor-4, Nor-5, Nor-6, and SSP-4 by maximum diversity (Figure 5E). The normal group was the main part in the maximum diversity. The intestinal flora of the normal group was more diverse at the OTU level before establishment of colitis, but the diversity of the intestinal flora was affected after establishment. The DSS samples showed that the intestinal flora was significantly different from that of the normal group at the OTU level in the most dissimilar. In order to understand the important role of intestinal microbiota in colitis mice, we used picrust 10 (community phylogenetic studies by reconstructing unobserved states) to predict high-throughput sequencing data based on 16S RNA. We obtained the clusters of orthologous groups (COG) atlas of microorganisms, and the results showed that this microbial community was mainly involved in carbohydrate transport and metabolism, general functional prediction, signal transduction mechanism, and cell wall membrane biogenesis, and was mainly related to cell metabolism and cell transfer activities (Figure 5F). The results revealed that SSP effectively controlled the relationship in the crosstalk between inflammatory DCs and gut microbiota in DSS-induced colitis.

## Discussion

The therapeutic effect of SSP on IBD has been confirmed in clinical research; however its mechanism of action is still ambiguous. In the present study, we demonstrated that SSP had a significant effect on experimental colitis, demonstrated by a reduction in body weight loss, shortening of colon length, decreased index of colonic weight and histological injury score, and restored pathological colonic damage.

IBD is a chronic inflammatory disease of the colon with high incidence, which results from many pathogenic factors. Although there is no cure for IBD currently, evidence indicates that dysbiosis of gut microbiota sustained by inflammation and injury are key factors in the pathogenesis (Chu et al., 2016). The composition and abundance of gut microbiota in IBD patients are markedly different from those in healthy people (Ding et al., 2018). In addition, an increase in the number of pathogenic bacteria and reduction in probiotics are important characteristics of IBD (Ananthakrishnan et al., 2018). In this study, we used DSS to induce colitis, and the immunopathological and inflammatory processes in mice are similar to those in human IBD (Malik et al., 2018). The gut microbiota of mice with DSS-induced colitis are imbalanced (Nanjundappa et al., 2017), which means that this model was suitable for the present study. The coverage curves revealed that most of the diversity was captured in all samples, and the microbial diversity in the DSS and DSS+SSP groups was lower than that in the normal group. PLS-DA revealed significant distances among the four groups, and the distance between the SSP and normal groups was the least. Our results also revealed that the DSS group compared with the normal group had an increased abundance of *Lachnospiraceae_NK4A136_group*, which belong to *Firmicutes* (Vila et al., 2018), and a decreased abundance of *Akkermansia* and *Corynebacterium*, as well as decreased bacterial alpha diversity, similar to previous studies (Zhou, 2017). After SSP administration in DSS-induced colitis mice, the abundance of these bacteria tended to return to normal. This indicates that SSP regulates homeostasis of the intestinal microbiota. Many studies have shown that modulation of the gut microbiota is a promising therapeutic approach for IBD (Ridaura et al., 2018). *Lachnospiraceae* and *Akkermansia* inhibited the development of colitis by influencing the level of cytokines or intestinal immune signaling (Li M. et al., 2019; Li P. et al., 2019). These results imply that SSP modulates gut microbiota composition and selectively promotes the protective strains to relieve DSS-induced colonic mucosal injury.

DCs are a critical immune cell subset bridging the innate and adaptive immune responses, which confront and capture antigens, and then migrate to a lymphoid organ, to promote T cell immune responses. This leads to the activation of antigen-specific T cells, eventually resulting in inflammation. In flaring IBD patients, the frequency and number of DCs in peripheral blood and the inflamed intestinal mucosa were increased when they were compared to healthy controls. These DCs generally display an activated phenotype defined by the increased expression of co-stimulatory molecules, and produce inflammatory cytokines (TNF-α, IL-6, and so on; Baumgart et al., 2011). As a well-established model for studying human colitis, at early stages of DSS-induced colitis, DC activation is now considered a direct trigger after epithelial damage, sufficient to trigger production of chemokines and pro-inflammatory cytokines. Some studies indicated that adoptive transfer of bone marrow-derived DCs exacerbated DSS-induced colitis in mice, while selective genetic ablation of DCs attenuated the severity of colitis. This hinted that DCs are critical in pathogenetic process of DSS-induced colitis, and anti-DC therapy can effectively block inflammatory cells infiltration to the inflamed colon and inhibit inflammation to protect against DSS colitis (Berndt et al., 2007; Massafra et al., 2015; Jo et al., 2018).

It has been shown that inflammatory DCs can induce Th1 and Th17 cell response, inhibit the T regulatory cell function, and lead to imbalance between pro-inflammatory and anti-inflammatory factors, as well as induce epithelial cell damage and colitis (Wang et al., 2018). DCs can also take part in the intercellular balance of Th1/Th2 cells to regulate the mucosal immune state by affecting or secreting cytokines (Qingzhen et al., 2012). We found that the concentrations of IL-1β, IL-4, IL-9, and IL-17A in the DSS group were significantly higher than those in the normal group. Expression of CD11c^+^CD103^+^E-cadherin^+^ and CD11c^+^CD103^+^TNF-α^+^ cells was increased by DSS. SSP and 5-ASA significantly reduced IL-1β, IL-4, IL-9, and IL-17A concentrations in the colitis mice, followed by lower expression of the CD11c^+^CD103^+^E-cadherin^+^ and CD11c^+^CD103^+^TNF-α^+^ cells. It is reported that IL-4 can induce differentiation of naïve CD4^+^ T cells into Th2 cells, and Th2 cells secrete IL-9 that acts as a pro-inflammatory factor that affects intestinal mucosal integrity by impairing intestinal barrier function (Bieber et al., 2017). In addition, IL-9 can induce differentiation of cells into Th9 cells in the presence of IL-4, and IL-17 is also a characteristic cytokine of Th17 cells (Cho et al., 2018). TNF-α plays an important role in inflammatory cell activation and epithelial cell permeability. E-cadherin plays an important role in maintaining intestinal epithelial barrier function (Goswami and Kaplan, 2011). So, we conclude that SSP inhibits inflammatory DCs to restrain expression of pro-inflammatory factors to relieve DSS-induced colonic mucosal damage. We also found that SSP improved the composition and abundance of gut microbiota in colitis mice. However, it has not been confirmed whether SSP regulates the gut microbiota in IBD. It is known that the gut microbiota play an important role in the pathological process of IBD, and is closely related with DCs, which are activated by bacterial antigens. However, it is unclear whether SSP regulates the crosstalk between the gut microbiota and inflammatory DCs. Therefore, our study was designed to investigate whether SSP can ameliorate IBD by regulating the interaction of inflammatory DCs and gut microbiota. As a classic inflammatory DCs, in the Figure 5C, we found that it was the similar levels of CD11c^+^CD103^+^E-cadherin^+^ cells when the different species level in normal and DSS+SSP groups, and which were obviously lower than in the DSS group. The results had hinted that obvious species differentiation of normal and DSS+SSP groups did not stimulate the proliferation of CD11c^+^CD103^+^E-cadherin^+^ cells, or SSP administrations led to some probiotics development to inhibit CD11c^+^CD103^+^E-cadherin^+^ cells hyperplasia and protected colonic mocusa from colitis mice.

In terms of gut microbiota in our study, *Akkermansia* and *Corynebacterium* were significantly reduced in the DSS group, which suggests that imbalance of gut microbiota is a significant pathological characteristic in DSS-induced colitis. After treatment of colitis with SSP for 7 days, *Akkermansia* and *Corynebacterium* were increased and *Lachnospiraceae_NK4A136_group* was reduced. This showed that SSP regulated the balance of gut microbiota in experimental colitis. A moderate enrichment of *Akkermansia* was identified in SSP-treated group compared with DSS treatment alone, suggesting a protective effect of SSP on *Akkermansia*. The possible mechanism underlying the therapeutic effect is because the anti-inflammatory activity of SSP helps to preserve the intact physiological contour of colonic lumen and host mucosa by preventing the mucin degradation, which was observed in the present study. Since mucin degradation was involved in bacterial pathogenesis of the colonic ecosystem, the mucus layer preservation would be beneficial for microbiota homeostasis in the lower gut (Miller and Hoskins, 1981). More importantly, the enrichment of *Akkermansia* is strongly associated with the mucus layer that demonstrated a promising therapeutic effect of SSP in the DSS-induced mouse colitis model. In addition, it is reported that the gut microbiota can stimulate expression of T cells and inhibit inflammatory cytokines, which play a key role in maintaining intestinal homeostasis, to induce proliferation, activation, or maturation of DCs. These DCs finally form inflammatory DCs, which show high expression of E-cadherin and TNF-α and secretion of pro-inflammatory factors. The enrichment of *Lachnospiraceae*_NK4A136 (around 3.5 folds) was identified in the DSS group compared with the normal group (around 3.0 folds of increase). Findings from current studies of *Lachnospiraceae*_NK4A136 abundance in IBD model are still a controversy (Xiao et al., 2016; Liu et al., 2018; Li M. et al., 2019; Li P. et al., 2019). Some of them illustrated that an increased abundance of *Lachnospiraceae*_NK4A136 contributes to alleviate inflammation in IBD model; however, that is not supported by our observation in the present study (Liu et al., 2018; Li M. et al., 2019; Li P. et al., 2019). That may need future study to address. The possible underlying reason is that the bioactive constitutes derived from SSP have promising regulatory effects on the colonic microbiota distribution, but no prebiotic activity. The prebiotic property mainly depends on a high content of indigestible polysaccharides such as fiber and resistant starch. Due to the limited content of indigestible polysaccharides, SSP supplementation was not shown to promote the growth of *Lachnospiraceae*_NK4A136, which normally ferment plant polysaccharide to produce short-chain fatty acids enhancing gut health.

It is reported that increased gut microbiota and mucin degradation can affect activation of inflammatory responses by binding to TLRs (Mehta et al., 2015). TLRs are important pattern recognition receptors on the membrane of DCs, and recognize bacterial antigen. Mature DCs present these bacterial antigens to T cells and activate T cells and induce cell-mediated immune response, and abnormal activation of T cells produces many pro-inflammatory factors that damage colonic mucosa. Our previous study showed that SSP inhibited expression of co-stimulatory molecules CD40/CD40L on DCs, and this limited the function of DCs in colitis (Smolinska et al., 2016). Furthermore, the efficient composition of SSP restrained pathogenic bacteria (*Enterococcus*, *Enterobacterium*, *Escherichia coli*, and *Enterococcus faecalis*), and produced beneficial bacterium (*Bifidobacterium* and *Lactobacillus*) *in vitro* (Tao et al., 2016). A decrease of population of pathogenic bacteria such as *E. coli* leads to preventing LPS to trigger the pro-inflammatory signaling events in DCs. This has been supported by the observation of our present study that the therapeutic effect of SSP is positively correlated with decreased level of CD11c^+^CD103^+^E-cadherin^+^ cells (inflammatory DCs), lower abundance of pathogenic bacteria, and increased abundance of beneficial bacterium. So, we conclude that the whole SSP or its effective component should regulate the structure and abundance of gut microbiota to control inflammatory DCs to improve inflammatory colonic injury.

In conclusion, the present study demonstrated that SSP effectively treated DSS-induced colitis, which may have been mediated by regulating interaction between inflammatory DCs and gut microbiota. The results suggest that SSP has therapeutic potential for IBD by targeting the gut microbiome and inflammatory DCs. However, it is unclear whether SSP regulates inflammatory DCs to alleviate IBD by interaction with gut microbiota and/or its metabolites, or some signaling pathways. This will be investigated in our future study.

## Data Availability Statement

The datasets generated for this study can be found in NCBI (accession: PRJNA637805).

## Ethics Statement

The animal study was reviewed and approved by Institutional Animal Care and Use Committee (IACUC) of Jiangxi University of Traditional Chinese Medicine. Written informed consent was obtained from the owners for the participation of their animals in this study.

## Author Contributions

FC and Y-TY contributed equally to this work as co-first authors. Conceived and designed the experiments by D-YL and H-MZ. Performed the experiments by FC, Y-TY, H-MZ, H-YW, Y-BZ, and JL. Contributed reagents, materials, analytical tools by D-YL and H-MZ. Analyzed the data by D-YL and FC. Wrote the paper by FC, H-MZ, and D-YL. All authors contributed to the article and approved the submitted version.

### Conflict of Interest

The authors declare that the research was conducted in the absence of any commercial or financial relationships that could be construed as a potential conflict of interest.
